# Characterization of the Bottlenecks and Pathways for Inhibitor Dissociation from [NiFe] Hydrogenase

**DOI:** 10.1021/acs.jcim.4c00187

**Published:** 2024-05-10

**Authors:** Farzin Sohraby, Ariane Nunes-Alves

**Affiliations:** Institute of Chemistry, Technische Universität Berlin, Straße des 17. Juni 135, 10623 Berlin, Germany

## Abstract

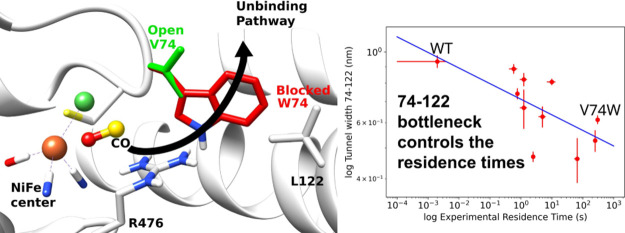

[NiFe] hydrogenases can act as efficient catalysts for hydrogen oxidation and biofuel production. However, some [NiFe] hydrogenases are inhibited by gas molecules present in the environment, such as O_2_ and CO. One strategy to engineer [NiFe] hydrogenases and achieve O_2_- and CO-tolerant enzymes is by introducing point mutations to block the access of inhibitors to the catalytic site. In this work, we characterized the unbinding pathways of CO in the complex with the wild-type and 10 different mutants of [NiFe] hydrogenase from *Desulfovibrio fructosovorans* using τ-random accelerated molecular dynamics (τRAMD) to enhance the sampling of unbinding events. The ranking provided by the relative residence times computed with τRAMD is in agreement with experiments. Extensive data analysis of the simulations revealed that from the two bottlenecks proposed in previous studies for the transit of gas molecules (residues 74 and 122 and residues 74 and 476), only one of them (residues 74 and 122) effectively modulates diffusion and residence times for CO. We also computed pathway probabilities for the unbinding of CO, O_2_, and H_2_ from the wild-type [NiFe] hydrogenase, and we observed that while the most probable pathways are the same, the secondary pathways are different. We propose that introducing mutations to block the most probable paths, in combination with mutations to open the main secondary path used by H_2_, can be a feasible strategy to achieve CO and O_2_ resistance in the [NiFe] hydrogenase from *Desulfovibrio fructosovorans*.

## Introduction

The hydrogenase family of enzymes are key for H_2_ transformation in many microorganisms, and they have recently attracted attention due to their ability to act as efficient catalysts to oxidize hydrogen and produce biofuel (H_2_ ⇌ 2 H^+^ + 2 e^–^) or even act as part of light-driven production pipelines of H_2_ through water splitting.^1−6^ However, some of the members of this family of enzymes are inhibited or irreversibly damaged and destroyed by gas molecules present in the environment, such as O_2_ and CO.^7−10^ Therefore, efforts have been made to develop strategies to rectify this problem and achieve inhibitor-tolerant enzymes.^11−22^ One possible strategy to achieve inhibitor-tolerant enzymes can be blocking the access of these inhibitors to the catalytic site by designing mutant forms through tunnel engineering.^16,23−30^ The difference in size and dipole moment between the substrate and inhibitor molecules of the hydrogenases suggests that this strategy is feasible. Tunnel engineering can be used to change the preferences of an enzyme for binding to and accommodating specific ligands by site-specific point mutations.^24,26,31,32^ In hydrogenases, the active site is buried in the core of the enzyme, and ligands need to travel a long distance through the tunnels to reach it. In 2005, Buhrke *et al.*(33) reported that an oxygen-tolerant hydrogenase from *Ralstonia eutropha* H16 can become sensitive to O_2_ by introducing specific point mutations that expand the tunnels leading to the active site, which ultimately facilitate the access of O_2_. This is evidence that tunnel engineering is a feasible strategy to achieve CO- and O_2_-tolerant hydrogenases.^33^

**Figure 1:**
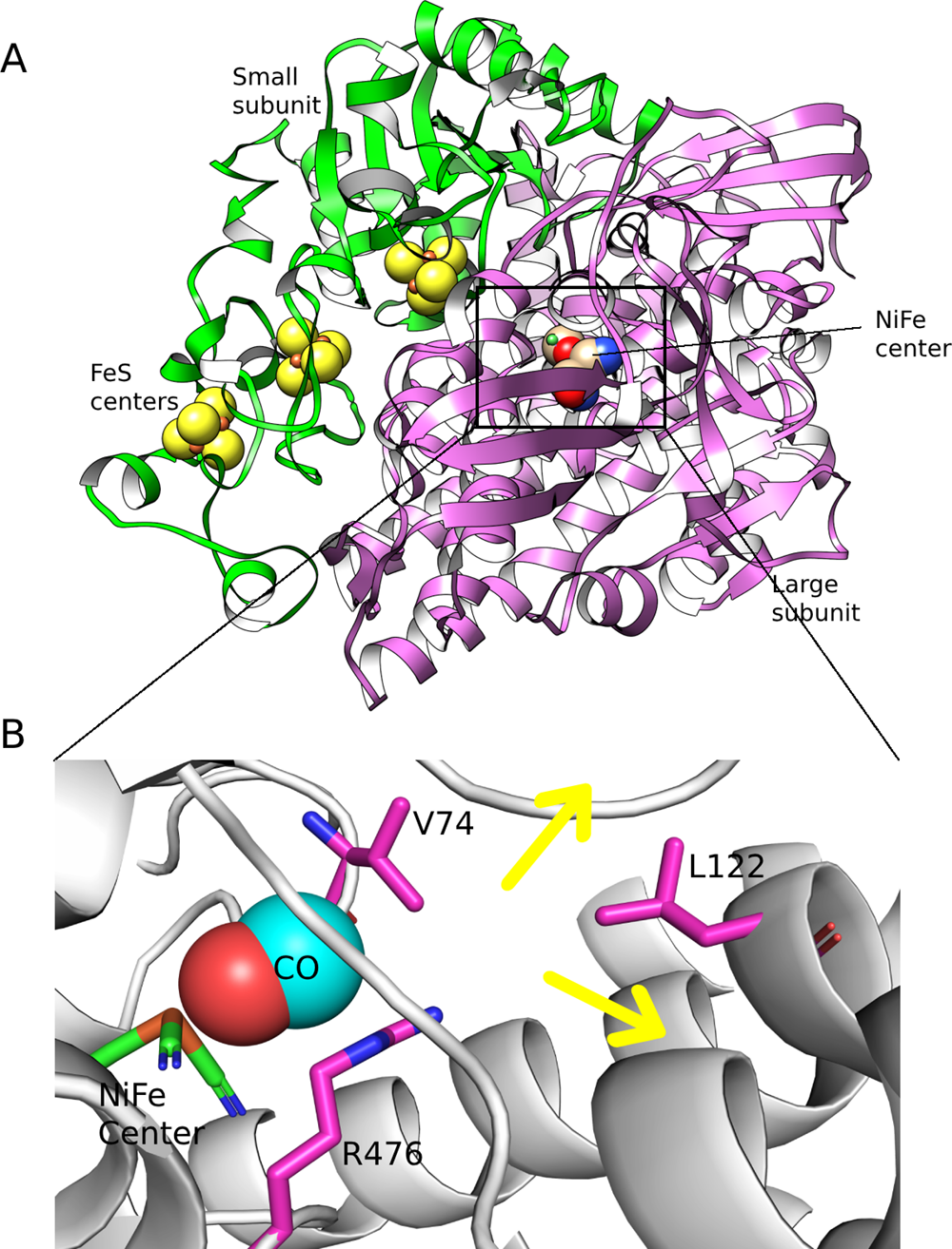
Structure of [NiFe] hydrogenase (PDB1YQW).^35^ (A) Small (green ribbon) and large (pink ribbon) subunits, and the positions of the metal centers (3 FeS centers in the small subunit and the active site in the large subunit). The metal centers and the active site are represented as spheres. (B) Active site of [NiFe] hydrogenase. Selected residues and metals of the active site are shown in stick representation, and CO is shown with spheres. Previous works suggested that two pairs of residues (74 and 122, and 74 and 476) were the main bottlenecks for gas diffusion. The two yellow arrows indicate the two major unbinding routes identified in this work.

Leroux *et al.* introduced protein film voltammetry, an experimental method that enabled the study of kinetics of binding and release of CO and other gas molecules from [NiFe] hydrogenases in a quantitative manner.^29^ Using this technique, they quantified the diffusion of CO, H_2_, and O_2_ inside the [NiFe] hydrogenase from *Desulfovibrio fructosovorans* (Figure 1A).^34^ In the work of Liebgott *et al.*, point mutations in this [NiFe] hydrogenase were introduced with the goal of understanding how changes in the structure of the tunnels can modulate the diffusion of gas molecules.^14,34^ They created 10 different mutants, with mutations at positions V74 and/or L122 of the large subunit (Figure 1B). Such residues were chosen because inspection of the crystal structure led to the hypotheses that the distance between these two residues was the main bottleneck for gas diffusion.^29,34^ Indeed, they observed changes in the kinetic rates of the inhibitors for binding and unbinding by orders of magnitude.^34^ Although the mutants delayed the binding of the inhibitors to the catalytic site, none of the mutants were tolerant to the inhibitors.

Understanding the role of mutations in the diffusion of the ligands through the tunnels and how they change the binding and unbinding pathways can give us insights on how to engineer inhibitor-tolerant mutants. Such pathways cannot be observed experimentally, but they can be studied using computational methods. One of the computational approaches to observe binding and unbinding pathways is molecular dynamics (MD) simulations. MD employs Newton’s laws to propagate the motions of atoms in a system, allowing one to investigate the motions of biomacromolecules.^36^ However, one of the main limitations of this method has been the time scales that can be achieved. Binding and unbinding events of small molecules happen in the millisecond time scale or slower, while conventional MD (cMD) simulations are usually limited to tens of microseconds.^37^ Therefore, it is not feasible to simulate binding events with cMD. In recent years, many enhanced sampling methods have been developed that can be employed to sample binding and unbinding events in the time scales achieved by cMD.^37−40^ τRAMD (τ-random accelerated molecular dynamics) is an enhanced sampling method in which a force of constant magnitude and random orientation is applied to the center of mass (COM) of the ligand molecule, increasing the chances of observation of unbinding events.^41^ τRAMD provides relative residence time (RT) values, which can be used to distinguish slow unbinding ligands from fast unbinding ligands and rank them accordingly. τRAMD has been used to investigate ligand unbinding in several systems, such as T4 lysozyme mutants, kinases, and heat shock protein 90, and it was able to reproduce experimental kinetic rates.^42−45^

Several works have studied the [NiFe] hydrogenase by computational methods, providing mechanistic insights about the diffusion of gas molecules inside the tunnels.^46−56^ Wang *et al.*(47,51) developed a master equation for calculation of gas diffusion rates within the [NiFe] hydrogenase and used their method to further understand gas diffusion in the mutants designed by Liebgott *et al.*(34) Based on their results, they proposed that in addition to the distance between residues 74 and 122, the distance between residues 74 and 476 (Figure 1B) is also a bottleneck that controls gas diffusion in the [NiFe] hydrogenase of *Desulfovibrio fructosovorans*. Additionally, they also proposed that mutations in the position 476 could lead to resistance to CO and O_2_. However, such a proposition could not be tested because R476 is essential for the catalytic activity of [NiFe] hydrogenase.

In this work, we focused on the wild-type (WT) form and 10 mutants of [NiFe] hydrogenase from *Desulfovibrio fructosovorans* reported by Liebgott *et al.*(34) We employed the enhanced sampling method τRAMD to obtain unbinding events of the substrate (H_2_) and inhibitors (O_2_ and CO) in order to understand the mechanism of diffusion of these gas molecules through the 30 Å long tunnels of this enzyme. The relative RTs computed with τRAMD for CO are in agreement with the experimental ones. We found that the RT is mainly controlled by the bottleneck between residues 74 and 122 (Figure 1B). We computed pathway probabilities for the unbinding of different gas molecules, and we observed that while the most probable pathways are the same for different gas molecules and different mutants, the secondary pathways can be different. Finally, we propose that blockage of the main paths in combination with opening of the main secondary path used by H_2_ can be a feasible strategy to achieve CO and O_2_ resistance in the [NiFe] hydrogenase from *Desulfovibrio fructosovorans*.

## Computational Methods

There are a total of 10 [NiFe] hydrogenase mutants that have experimental kinetics data determined for the unbinding of CO (Table S1), and 4 mutants with experimental kinetics data determined for the unbinding of O_2_.^34^ Additionally, kinetics data are available for CO unbinding from the WT [NiFe] hydrogenase. For the substrate (H_2_), there are only experimental Michaelis constants available.^34^ We studied a total of 13 complexes, the unbinding of CO from 10 [NiFe] hydrogenase mutants and from the WT [NiFe] hydrogenase and also the unbinding of O_2_ and H_2_ from the WT [NiFe] hydrogenase (Table S1). Since experimental kinetics data were not available for the unbinding of O_2_ or H_2_ from most of the mutants, we only investigated the unbinding of O_2_ and H_2_ from the WT [NiFe] hydrogenase.

The WT [NiFe] hydrogenase from *Desulfovibrio fructosovorans* and the mutants V74M and V74M-L122M had crystal structures available (PDB IDs 1YQW,^35^ 3H3X,^57^ and 3CUR,^29^ respectively). The structures were used to model the protein–ligand complex. The peroxide ion in the structures was replaced by the gas molecule simulated (H_2_, CO, or O_2_). In the case of CO, the O atom was put close to the [NiFe] metal center. For the rest of the mutations, the crystal structure of the WT [NiFe] hydrogenase was used as a starting model, and the rotamer tool in UCSF chimera software^58,59^ was used to make point mutations on the 74 or 122 positions of the large subunit of the enzyme. Then, the protonation states of the residues for all mutants at pH 7, the pH used for measuring experimental kinetic rates,^34^ were determined using Propka version 3.5.2,^60−62^ as implemented in the program pdb2pqr version 2.1.1.^63,64^

All MD simulations were carried out using GROMACS-RAMD version 2.0^41,65^ and the AMBER99SB force field.^66^ In order to describe the metal sites of the [NiFe] hydrogenase, the force field bonded parameters and the partial charges of the metal centers were obtained from the works of Smith *et al.*(46) and Teixeira *et al.*,^67^ respectively. The parameters were selected based on the state of the metal centers. We selected the reduced state for the [FeS] center and the NiB state for the [NiFe] center. The force field parameters of the gas molecules (H_2_, CO, O_2_) were obtained from the literature or from quantum mechanical (QM) calculations. For O_2_ and H_2_, the bonded parameters, Lennard-Jones parameters, and partial charges were obtained from Wang *et al.*(68) For CO, the bonded parameters and Lennard-Jones parameters were obtained from the work of Straub *et al.*(69) For the partial charges of CO, we performed QM calculations using Gaussian,^70^ Hartree–Fock, and the 6-31G* basis set, which resulted in partial charges of +0.059 e for C and −0.059 e for O (Table S2).

The protein–ligand complex was placed in the center of a cubic box with a distance of 1 nm from all edges and solvated with the TIP3P^71^ water model. Then, sodium and chloride ions were added to produce an ionic strength of 118 mM. The ionic strength was adopted to reproduce the conditions used for the protein film voltammetry experiments to obtain kinetic rates.^34^ The final systems had ∼113,000 atoms.

Next, we performed energy minimization and a 50 ns cMD simulation for each starting structure (details below). The corresponding backbone RMSD values can be found in Figure S1. The gas molecules were positionally restrained with a harmonic force constant of 5000 kJ/mol^–1^ nm^–2^ in order to keep them inside the active site. Then, the end frame of the 50 ns cMDs was used to run five replicas of cMD, each with a duration of 20 ns. The five replicas were performed to increase diversity among the structures. The end frame of each replica was then used as the starting structure of the τRAMD runs. For each replica, we performed 15 τRAMD simulations to achieve a total of 75 dissociation events for each mutant. The RT value in one trajectory was calculated as the time it takes for the gas molecule to reach the unbound state, which was defined as the state when the gas molecule has no contacts with the protein. The number of contacts between the gas molecule and the protein was calculated using a threshold of 6 Å for atomic distances. All atoms were considered.

The starting structure was energy minimized using the steepest descent algorithm until the maximum force was less than 10 kJ mol^–1^ nm^–1^. Then, the system was heated to 310 K using the Berendsen thermostat.^72^ Next, the pressure was equilibrated to 1 bar using the Berendsen barostat.^72^ After temperature and pressure equilibration, additional steps were performed to reduce the positional restraints over the system’s heavy atoms in four steps (500, 200, 50, and 0 kJ/mol^–1^ nm^–2^, but 5000 kJ/mol^–1^ nm^–2^ positional restraints on the ligand atoms were kept in all simulations, except the τRAMD unbinding simulation runs). In all simulations, after equilibration, temperature and pressure coupling were achieved with the Nose–Hoover thermostat^73,74^ and the Parrinello–Rahman barostat, respectively.^75,76^ The covalent bonds to hydrogen atoms were constrained using the Linear Constraint Solver (LINCS) algorithm to maintain constant bond lengths.^77^ Bond lengths for the solvent were constrained using the SETTLE algorithm.^78^ The long-range electrostatic interactions were treated using the particle Mesh Ewald (PME) method with a real-space cutoff of 1.2 nm, PME order of 4, and a Fourier grid spacing of 1.2 Å.^79,80^ van der Waals forces were computed using a cutoff of 1.2 nm. The magnitude of the force for the τRAMD runs was set to 1 kcal/molÅ, the threshold distance was set to 0.0025 nm, and the evaluation frequency was set to 100 fs. The procedure to choose the force magnitude will be explained in the results section.
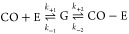
1

Eq 1 shows the process of association and dissociation of CO to/from the enzyme (E), as observed in the experiments of Liebgott *et al.*(34) In CO-E, CO forms a coordination complex with the [NiFe] center. The geminate state (G) represents the state where the gas molecule is close to the [NiFe] center but does not form a coordination complex with it. The *k*_out_ values derived from experiments^34^ represent the rate in which the gas molecule goes from the bound state (CO-E), where CO forms a coordination complex with the [NiFe] center, to the unbound state (CO + E), in which the CO molecule is free in the solvent. However, in our dissociation trajectories, since we are using a classical force field, we did not simulate the rupture of the coordination complex between CO and the [NiFe] center. The values obtained from simulations represent *k*_–1_. Previous work^34^ has presented and discussed evidence that changes in the *k*_out_ values are mainly due to changes in the *k*_–1_ values for the different mutants of [NiFe] hydrogenase studied by Liebgott *et al.*(34) and investigated here. Along the paper, we discuss the diffusion of gas molecules in terms of RT (1/*k*_out_ for experiments, 1/*k*_–1_ for simulations).

Analyses of dissociation trajectories were performed using GROMACS utilities and UCSF Chimera.^58^ Analysis of the tunnels was performed using CAVER 3.0 PyMOL Plugin, Pymol 2.0, and AQUA-DUCT 1.0.^81−83^ We used CAVER 3.0 PyMOL Plugin^81^ for tunnel identification in the crystallographic structure of [NiFe] hydrogenase (PDB 1YQW(35)). The coordinates of the COM of the CO molecule were used as the starting point. A minimum probe radius of 0.9 Å and a clustering threshold of 3.5 Å were used. Other settings were set to default values. The assignment of trajectories to tunnels was performed using AQUA-DUCT^82^ and visual inspection. First, AQUA-DUCT was used to trace the pathways of the gas molecule inside the tunnels in the 75 unbinding trajectories and cluster them together. We used the mean shift clustering algorithm with a bandwidth of 7. Other settings were set to default values. Then, by visually inspecting the clusters of pathways (traces and exit points), we assigned each cluster to one of the tunnels identified by CAVER. The traces and exit points for CO dissociation from the WT [NiFe] hydrogenase and from the 10 mutants can be found in Figures S2–S4. The Pymol session with the nine tunnels identified by CAVER and the AQUA-DUCT output Pymol session containing clusters of pathways for the WT [NiFe] hydrogenase are available as Supporting Information.

## Results and Discussion

### τRAMD can Discriminate Complexes with Short and Long RTs

The main parameter to be optimized in τRAMD is the magnitude of the random force applied on the COM of the gas molecule to enhance dissociation. The magnitude of the force has a direct effect on the speed of the unbinding process. If the force is too high, it will result in a very fast unbinding event, which could lead to reduced sampling of the transition state. We found that 1 kcal/molÅ of force magnitude is optimum for our case, providing a good compromise between force magnitude and computational time to sample unbinding events (Figure S5, Table S3). Moreover, the force of 1 kcal/molÅ provided a good discrimination between the unbinding rates of the fastest, WT-CO, and the slowest dissociating complex, V74W-CO (Figure S5, Table S3). We also tested different threshold distances (Figure S6, Table S4), which determine whether there will be a change in the orientation of the force according to the ligand displacement for a given time interval, and adopted the value of 0.0025 nm for the work.

**Figure 2:**
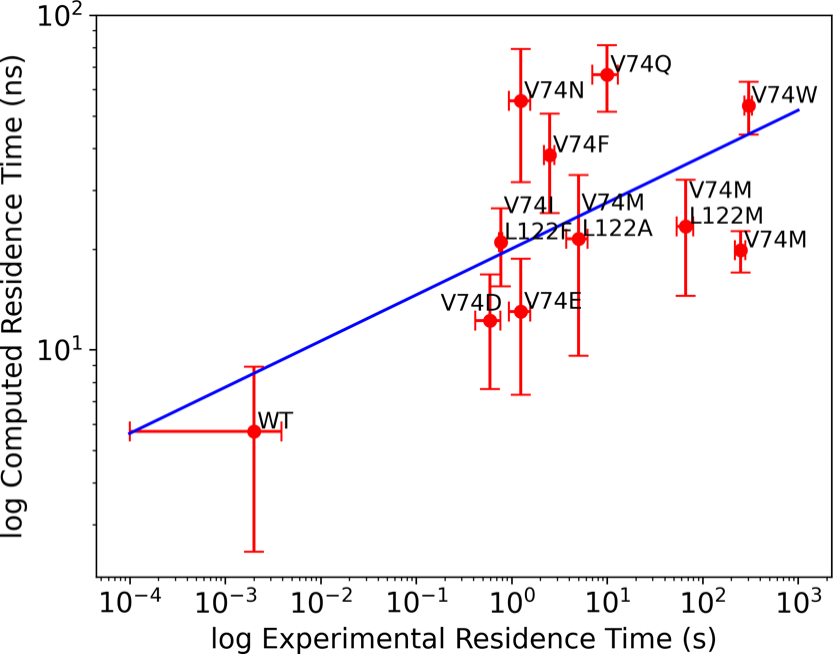
Comparison between experimental and computed RTs of CO in complex with WT or mutants of [NiFe] hydrogenase using τRAMD with 1 kcal/molÅ of force magnitude (*R* = 0.62, ρ = 0.57). If outliers (V74N and V74Q) are excluded, a higher correlation is achieved (*R* = 0.79, ρ = 0.75). Data can be found in Table S1. The blue line is a linear fit to the data (slope = 0.14), and the error bars represent standard deviations of the mean RT values for five replicas for each mutant.

Before the τRAMD unbinding simulation runs, we performed a 50 ns cMD run for every system to stabilize the conformation of the mutated residues and of the residues near the mutation. Then, we used the final snapshot to perform five 20 ns cMD (replicas) in order to explore the conformational space. The end frames of the five replicas were used as the initial structures for the subsequent τRAMD simulations. Figure 2 and Table S1 show the experimental and computed RT values for CO in complex with WT and 10 different mutants. We achieved a Pearson correlation coefficient (*R*) of 0.62 and a Spearman’s rank correlation coefficient (ρ) of 0.57. The *R* and ρ values are reasonable and allow one to discriminate complexes with long and short RTs. It is worth mentioning that, if outliers (V74N and V74Q) are excluded, an *R* of 0.79 and a ρ of 0.75 are achieved. However, we also note that the *R* value obtained is dependent on the WT data point (*R* of 0.62 and 0.29 in the presence and absence of the WT, respectively, Table S5), while the ρ value is less dependent on the WT data point (ρ of 0.57 and 0.43 in the presence and absence of the WT, respectively, Table S5). Taken together, the data show that the ranking provided by the relative RTs computed with τRAMD is in agreement with experiments. Data analysis and discussion refer to all data points presented in Figure 2.

The slope value obtained in our work for the comparison of computed and experimental RTs, 0.14 (Figure 2), is lower than the slope value of 0.47, obtained in a previous work which investigated dissociation of CO from [NiFe] hydrogenase,^51^ indicating a lower sensitivity to discriminate complexes with short and long RTs. One possible explanation for this difference is the fact that we computed relative RTs, while the previous work computed absolute RTs. A slope of 1 is expected in cases where experimental and computed absolute RTs are compared. Another important difference is the number of complexes investigated: the previous work investigated 4 complexes, 3 of them with experimental structures available, while we investigated 11 complexes, for which only 3 had experimental structures available. The modeling of mutants without experimental structure introduces more uncertainty and could have contributed to a lower slope value. Additionally, bulky mutations could have introduced allosteric changes or caused large structural changes in the enzyme, effects that would probably not be captured in our simulations.

Previous works^42−45^ used τRAMD to compute relative RTs for complexes between proteins and small drug-like molecules, achieving coefficients of determination (*R*^2^) from 0.78 to 0.94. Here, we obtained an *R*^2^ value of 0.39 (or 0.62 without outliers, Table S5). This lower performance, in comparison to previous works, can be partially explained by the challenge of representing metal sites and small gas molecules using a classical force field, with fixed point charges. Another challenging aspect of the current work is that the differences in RTs for the mutants come from differences mainly in the transition states, as indicated by the presence of a high correlation between experimental *k*_on_ and experimental *k*_off_ values (Figure S7). Classical force fields may be less sensitive to differences in transition states, which are higher in energy and less stable, in comparison to differences in bound states.

### The Bottleneck between Residues 74 and 122 Modulates RTs for CO

**Figure 3:**
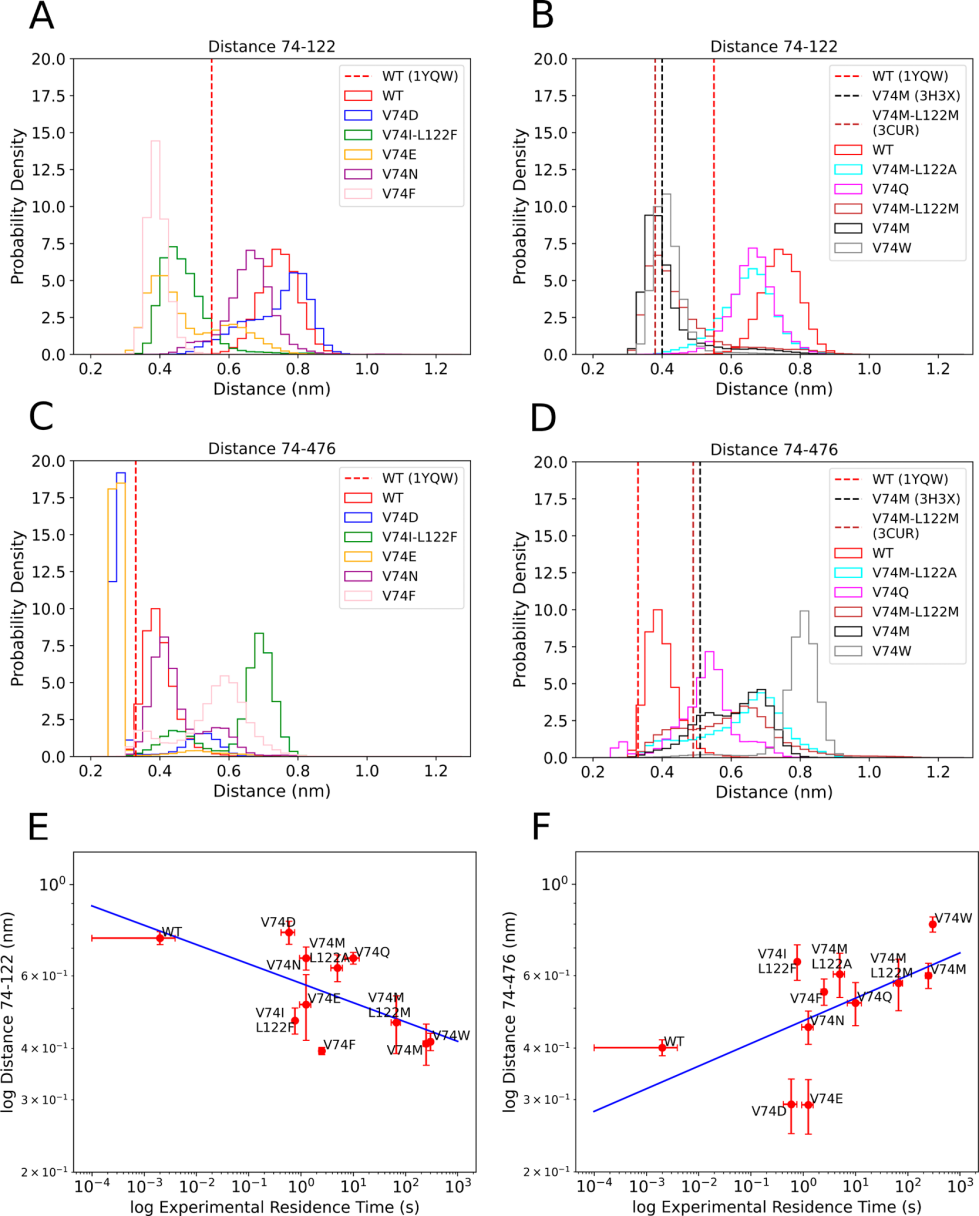
Distribution of the minimum distances between the terminal heavy atoms of the residues governing the two bottlenecks and correlation with experimental RTs. Distribution of the minimum distances between the residues 74 and 122 in different mutants of [NiFe] hydrogenase (A) in fast unbinding mutants and (B) in slow unbinding mutants. The WT was included in both for better comparison. (C) Distribution of the minimum distances between the residues 74 and 476 in fast unbinding mutants and (D) in slow unbinding mutants of [NiFe] hydrogenase. The atoms used to calculate the distances can be found in Table S6. The distances between the residues governing the bottlenecks in the three crystallographic structures (PDB ID1YQW, 3H3X, and 3CUR for WT, V74M, and V74M-L122M, respectively) are shown as dashed lines. (E) Correlation between experimental RT values and the average distances between residues 74 and 122 in different mutants of [NiFe] hydrogenase (*R* = −0.64) and (F) correlation between experimental RT values and the average distances between residues 74 and 476 in different mutants of [NiFe] hydrogenase (*R* = 0.58). The distances between the residues of the bottlenecks were calculated using the entire τRAMD dissociation trajectories in all mutants. The blue lines are linear fits to the data, and the error bars represent standard deviations of the mean distances for 75 trajectories for each mutant.

As stated in the introduction, two bottlenecks were proposed previously as the main factors modulating the diffusion of gas molecules inside [NiFe] hydrogenase, the distance between residues 74 and 122 and the distance between residues 74 and 476. We calculated the width of these two bottlenecks by calculating the minimum distance between the terminal heavy atoms of the investigated residues (Table S6). The distribution of the distances is presented in Figure 3A–D. The distances found in the crystal structures are shown as dashed lines. For the 74–122 bottleneck, it can be seen that the distances found in the simulations fluctuate around the values found in the crystal structures for the mutants V74M and V74M-L122M, while the distances found in the simulations are larger than the distance observed in the crystal structure of the WT [NiFe] hydrogenase. This observation showcases the importance of considering dynamics and flexibility to investigate tunnels and ligand dissociation. The correlation between the width of the 74–122 bottleneck and RT is strongly negative, showing that the longer the RT, the narrower the width of the 74–122 bottleneck (*R* = −0.64, Figure 3E). This shows that the 74–122 bottleneck is effectively regulating RT values and CO dissociation in [NiFe] hydrogenase. This result is in disagreement with previous computational work from Wang *et al.*,^51^ which did not find a clear correlation between the width of the 74–122 bottleneck and the RT values for the mutants of [NiFe] hydrogenase. Possible explanations could be the longer simulations or the larger number of mutants considered in the present work.

The correlation between the width of the 74–476 bottleneck and RT is positive (*R* = 0.58, Figure 3F), showing that the longer the RT, the wider the width of the 74–476 bottleneck. Therefore, we found no evidence that the distance between residues 74 and 476 acts as a bottleneck for CO dissociation in the mutants investigated. We hypothesize that bulky residues at position 74 cannot fit properly in the free space available in the tunnel, and the positive correlation found is due to the bulkiness of the residues or, in the case of the M mutation, high fluctuations of the flexible residue.

In order to understand the diffusion of CO inside the tunnels before leaving them through the exit point, we calculated the time it takes for CO to lose contact with the residues at positions 74 and 122. We found that, on average, CO stays in the tunnels for about 4 ns after losing contact with residues 74 and 122 (Table S7). The variation in RT values in simulations of different [NiFe] hydrogenase mutants is mostly dictated by the time it takes for CO to pass the 74–122 bottleneck, which is another evidence of the importance of this bottleneck for modulating RT values in [NiFe] hydrogenase.

### Paths T1 and T2 are the Preferred Paths for Unbinding

**Figure 4:**
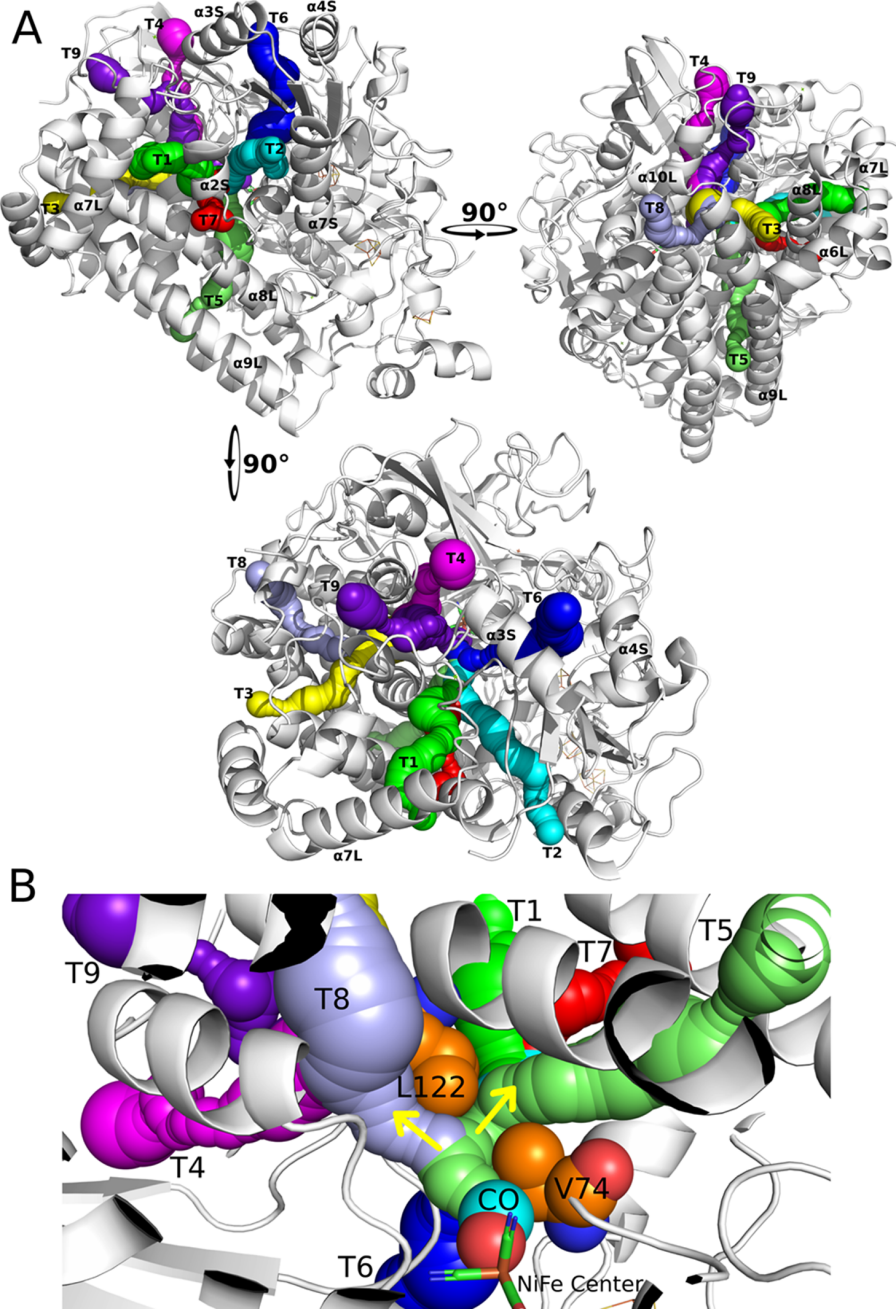
Tunnels and unbinding pathways for CO identified in the [NiFe] hydrogenase. (A) Nine tunnels (T1–T9) were identified inside the crystallographic structure of the WT [NiFe] hydrogenase (PDB ID1YQW)^35^ using the CAVER 3.0 Plugin in Pymol.^81,83^ The secondary structures are named according to order of appearance in the primary structure and subunits (S for small; L for large). Example: α2S, second α-helix from the small subunit. The gas molecules used these tunnels as unbinding pathways in the trajectories. (B) Course of the tunnels is divided into two major groups, as indicated by the yellow arrows. Note that T3, T4, T8, and T9 skip the 74–122 bottleneck, and T1, T2, T5, T6, and T7 go through the 74–122 bottleneck. Residues V74 and L122 are represented as orange spheres.

We mapped the tunnels connecting the active site to the solvent in the crystallographic structure of the WT [NiFe] hydrogenase (PDB ID1YQW)^35^ using CAVER 3.0^81^ and found nine different tunnels (Figure 4A). By analyzing the tunnels, we found that their starting points can be divided into two major groups. As it is shown in Figure 4B, most of the tunnels (T1, T2, T5, T6, and T7) go through the 74–122 bottleneck, while some tunnels (T3, T4, T8, and T9) skip the 74–122 bottleneck.

**Figure 5:**
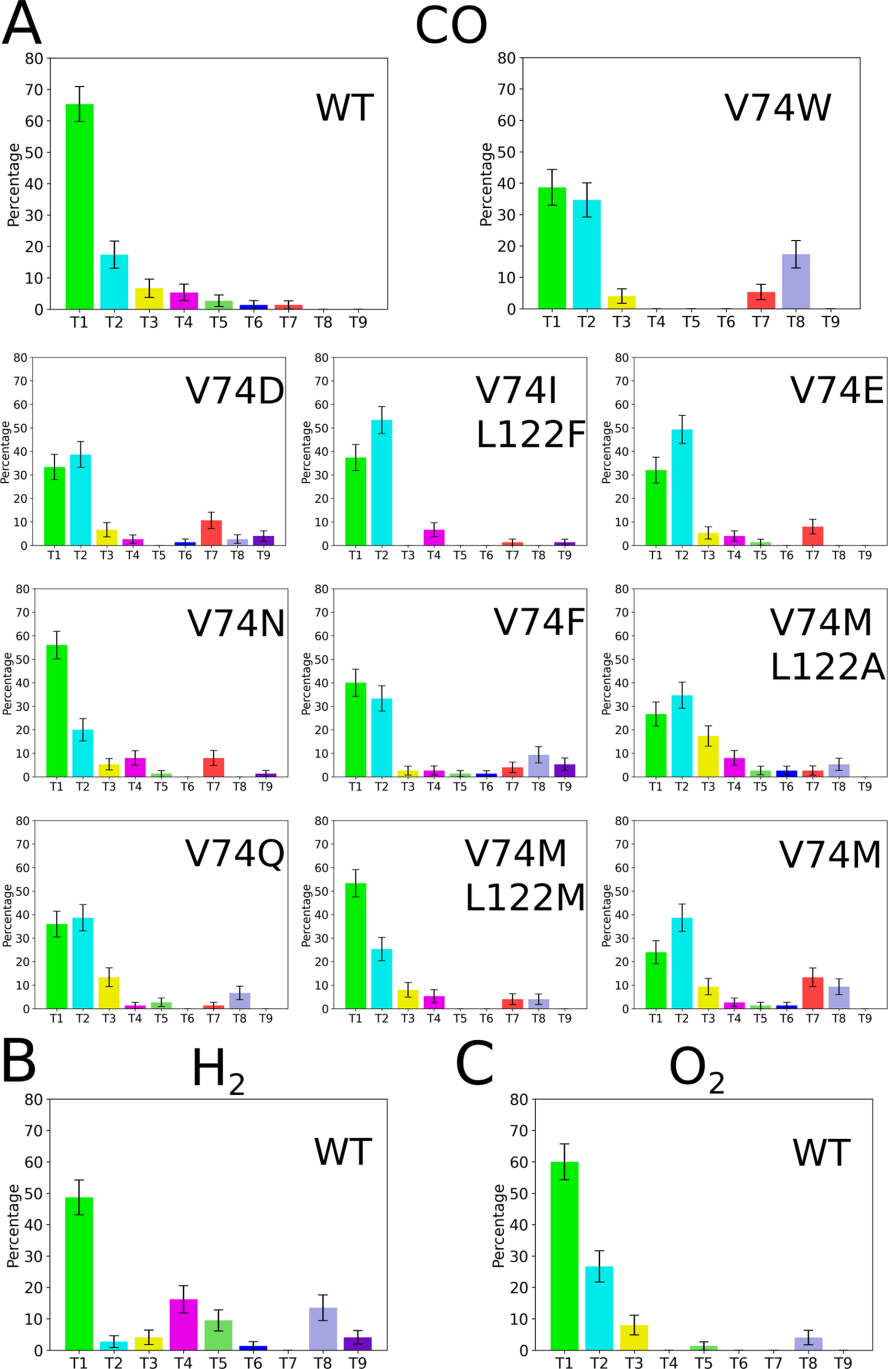
Populations of different pathways that CO, H_2_, and O_2_ use for unbinding from different mutants of [NiFe] hydrogenase. The colors of the paths match the colors of the associated tunnels in Figure 4A. The standard deviation on each bar comes from bootstrapping analysis. (A) CO complexes, WT-CO with the shortest RT, and the V74W-CO complex with the longest RT are shown on top. Below, the other CO mutant complexes are shown from the one with the shortest RT (up left corner) to the one with the longest RT (right down corner). (B) H_2_–WT and (C) O_2_–WT complexes. Unbinding pathways were obtained from τRAMD dissociation trajectories and identified using AQUA-DUCT 1.0.^82^

We identified nine different pathways by tracking the motion of CO in the unbinding trajectories using AQUA-DUCT 1.0.^82^ These nine paths from the trajectories are associated with the nine tunnels identified in the crystallographic structure, showing that CO can use all of these nine different tunnels for unbinding. Figure 5 shows the population of the unbinding pathways of CO in different mutants. The most frequently used pathways are through the T1 and T2 tunnels in all of the mutants. The T1 and T2 tunnels both end at either side of the second α-helix of the small subunit (α2S), and the route of both tunnels is the same until they reach this helix. Our results suggest that the exit points of T1, T2, and T7 are controlled by the α2S, α7L, and α7S α-helices (Figure 4A). It is interesting to note that in mutants with longer RTs and more restriction for CO diffusion, there is an increase in the utilization of secondary or alternative exit paths, such as path T8. It is also important to mention that CO can move between pathways and diffuse in the tunnels until ultimately fully getting out of the enzyme. The unbinding pathways and populations presented in Figure 5 are associated with the last path accessed by CO before it left the interior of the enzyme.

The pathways identified here are in qualitative agreement with previous works which investigated pathways of gas molecules inside the [NiFe] hydrogenase of*Desulfovibrio fructosovorans*. It has been reported that there is a “VA”-shaped set of gas tunnels connecting the catalytic site to the surface of the enzyme.^47,50^ This “VA”-shaped tunnel corresponds to tunnels T1, T2, T3, T5, and T7 characterized here. Wang *et al.*(47) reported that in addition to the “VA”-shaped tunnels, they have identified two more pathways for H_2_, O_2_, and CO to reach the catalytic site that skip the 74–122 bottleneck, which is in agreement with our results. Oteri *et al.*(48) investigated the diffusion pathways of H_2_ from the enzyme surface to the catalytic site using MD and Brownian dynamics simulations. They presented the five most frequent tunnels, which are consistent with the tunnels identified in our work. Additionally, Kalms *et al.*,^49^ using simulations of the [NiFe] hydrogenase from *Ralstonia eutropha*, reported two tunnels, A and B. Tunnel A corresponds to T1, T2, and T7, and tunnel B corresponds to T3, T5, and T8.

### Different Gas Molecules Use Different Secondary Paths for Unbinding

In addition to CO, we also performed τRAMD simulations to study the unbinding pathways of O_2_ and H_2_ from the WT [NiFe] hydrogenase (Table S1). We found that, similar to CO, paths T1 and T2 are the most frequently used pathways for O_2_ and H_2_ unbinding (Figure 5). However, the secondary paths used by the different gas molecules are different. While CO and O_2_ have a low probability of utilization of secondary pathways during unbinding from the WT [NiFe] hydrogenase (less than 20% population for paths different from T1 and T2), H_2_ uses paths T4 and T8 more frequently. Therefore, we propose that mutations to block the main paths (T1 and T2) such as V74W and V74F or L122W and L122F, in combination with mutations to open the main secondary path used by H_2_ preferentially, T4, such as the mutation of residues L534 or I24 to smaller residues, can be a feasible strategy to achieve CO and O_2_ resistance in the [NiFe] hydrogenase from *Desulfovibrio fructosovorans*. Another idea is the introduction of cysteine residues in the 74 and 122 positions to permanently block the 74–122 bottleneck with a disulfide bond. However, additional MD simulations would be useful to further investigate the effect of such mutations over the kinetic rates and binding paths of the three gas molecules.

The sizes of the different gas molecules explain why they have different preferences concerning secondary paths for unbinding. In the case of H_2_, the higher mobility and small size of the gas molecule explains why H_2_ can use pathways with low populations for the other gas molecules, like paths T4 and T8. We can also see this behavior for CO in the V74W, V74F, and V74Q mutants, in which the width of the 74–122 bottleneck is the shortest (Figures 3 and 5), and the ligand tries to exit the enzyme using uncommon pathways. O_2_ and CO have very similar pathway populations and are larger than H_2_. Therefore, we conclude that the size of the gas molecule is important in determining the secondary pathways used in the unbinding events.

## Conclusions

The hydrogenase family of enzymes are of technological importance since they can be used for clean energy production. However, some of the members of this family of enzymes that have high catalytic rates, such as the [NiFe] hydrogenase from *Desulfovibrio fructosovorans*, have been evolved in anaerobic environments, and exposure to gas molecules present in the atmosphere, such as O_2_ and CO, can inhibit or damage the catalytic site, limiting their use in biofuel cells. One strategy to get around this problem is to engineer this enzyme to be O_2_- and CO-resistant by introducing point mutations to block the access of inhibitors to the catalytic site. Herein, we studied the pathways for CO unbinding from 10 different mutants of [NiFe] hydrogenase using τRAMD. While previous works proposed the existence of two bottlenecks (residues 74–122 and residues 74–476) to control gas diffusion, we found evidence that only one of these bottlenecks, the 74–122 bottleneck, effectively modulates the dissociation rates of CO in the mutants simulated. We also identified nine different tunnels connecting the catalytic site to the surface of the enzyme. We found that while the most utilized paths for dissociation from the WT [NiFe] hydrogenase are the same for H_2_, CO, and O_2_, the secondary paths change for the different gas molecules, offering an opportunity for the rational design O_2_- and CO-tolerant mutants of [NiFe] hydrogenase. We propose that mutations to block the main paths, T1 and T2, in combination with mutations to open one of the main secondary paths used by H_2_, T4, can be a feasible strategy to achieve CO and O_2_ resistance in the [NiFe] hydrogenase from *Desulfovibrio fructosovorans*.

## Data Availability

Additional Supporting Information is available in the link below to ensure reproducibility of the results (sample input files to run molecular dynamics simulations; modified force field files, including parameters for the metal centers in [NiFe] hydrogenase and parameters for the gas molecules; structures used to start simulations; Pymol session with the nine tunnels identified, AQUA-DUCT output Pymol session for the WT [NiFe] hydrogenase): 10.5281/zenodo.11033626.

## Supporting Information Available

Experimental vs computed RT values using 1 kcal/molÅ of force magnitude for complexes simulated using τRAMD; force field parameters of gas molecules; backbone RMSD values for each [NiFe] hydrogenase mutant in the 50 ns MD runs; unbinding pathways of CO from the WT [NiFe] hydrogenase; description of the assignment of pathways to tunnels for the WT [NiFe] hydrogenase using AQUA-DUCT; unbinding pathways of CO from each mutant of [NiFe] hydrogenase; mean RT values and associated standard deviations for CO dissociation from [NiFe] hydrogenase in the tests using different force magnitudes; mean RT values and associated standard deviations for CO in complex with WT [NiFe] hydrogenase or with the V74M mutant using different values of threshold distance; Pearson's correlation coefficient (*R*), coefficient of determination (*R*^2^) and Spearman's rank correlation coefficient (ρ) of all data, data with outliers (V74N and V74Q) removed and data with WT removed; atom names of the residues used to calculate the distances for the 74–122 and 74–476 bottlenecks; estimated time of CO’s residency inside the tunnels without contacts with residues 74 or 122; and comparison between experimental *k*_in_ and experimental *k*_out_ values^1^ (PDF)
