# Supplementary material for: Characterization of the Bottlenecks and Pathways for Inhibitor Dissociation from [NiFe] Hydrogenase
Source: J Chem Inf Model. 2024 May 10;64(10):4193–203. doi: 10.1021/acs.jcim.4c00187 (PMC11134402; doi:10.1021/acs.jcim.4c00187)
Supplement: Supplementary file 1 — ci4c00187_si_001.pdf [file ci4c00187_si_001.pdf]

Supplementary Information

Characterization of the bottlenecks and pathways for inhibitor dissociation from [NiFe] hydrogenase

Farzin Sohraby, Ariane Nunes-Alves*
Institute of Chemistry, Technische Universität Berlin, Straße des 17. Juni 135, 10623 Berlin, Germany
*Corresponding author: ferreira.nunes.alves@tu-berlin.de

Experimental vs computed residence times using 1 kcal/molÅ

Table S1 shows the residence time (RT) values for the τRAMD simulations of CO's dissociation from multiple mutants using 1 kcal/molÅ of force. This set of τRAMD simulations was used for further analysis.

Table S1. Experimental vs computed residence time (RT) values using 1 kcal/molÅ of force magnitude for complexes simulated using τRAMD.

		experiments					simulations		
**ligand**	**mutation**	**k_{out} (s^{-1})[a]**	**STD (s^{-1})[b]**	**RT (s)[c]**	**STD (s)[d]**	**Ranking[e]**	**RT (ns)[f]**	**STD (ns)[g]**	**Ranking[h]**
CO	WT	500	490	0.002	0.0019	1	5.7	3.21	1
CO	V74D	1.7	0.5	0.59	0.17	2	12.22	4.6	2
CO	L122F V74I	1.3	0.05	0.77	0.03	3	20.98	5.51	5
CO	V74E	0.8	0.2	1.25	0.31	4	13.02	5.68	3
CO	V74N	0.8	0.2	1.25	0.31	5	55.52	23.86	10
CO	V74F	0.4	0.05	2.50	0.31	6	38.2	12.59	8
CO	L122A V74M	0.2	0.05	5.00	1.25	7	21.44	11.84	6
CO	V74Q	0.1	0.03	10.00	3.00	8	66.42	14.95	11
CO	L122M V74M	0.015	0.003	66.67	13.33	9	23.36	8.87	7
CO	V74M	0.004	0.0005	250.00	31.25	10	19.84	2.82	4
CO	V74W	0.0033	0.0003	303.03	27.54	11	53.62	9.62	9
H_2	WT	-	-	-	-	-	2.32	1.25	-

O_2	WT	-	-	-	-	-	8.98	2.84	-

a. Experimental mean k_{out} values obtained from the work of Liebgott et al[1].
b. Standard deviation values associated with the experimental mean k_{out} values.
c. Experimental mean residence time values calculated from the experimental k_{out} values ($1/k_{out}$).
d. Standard deviation values associated with the experimental mean residence time values.
e. Ranking of the experimental mean residence time values from the shortest to the longest values.
f. Computed mean residence time values from τRAMD simulations.
g. Standard deviation values associated with the computed mean residence time values.
h. Ranking of the computed mean residence time values

Table S2. Force field parameters of gas molecules.

	**Bond Parameters**		**LJ Parameters**		**Partial charges**
	**equilibrium bond length (Å)**	**harmonic force constant (kcal/molÅ^2)**	**sigma (σ) (Å)**	**epsilon (ε) (kcal/mol)**	
**H_2**	0.74[2]	Constrained	2.91[2]	0.015[2]	0[2]
**O_2**	1.20[2]	Constrained	3.29[2]	0.104[2]	
**C**	1.12[3]	Constrained	3.83[3]	0.026[3]	+0.059[a]
**O**			3.12[3]	0.159[3]	-0.059[a]

a. Calculated by Gaussian[4], Hartree Fock and the 6-31G* basis set.

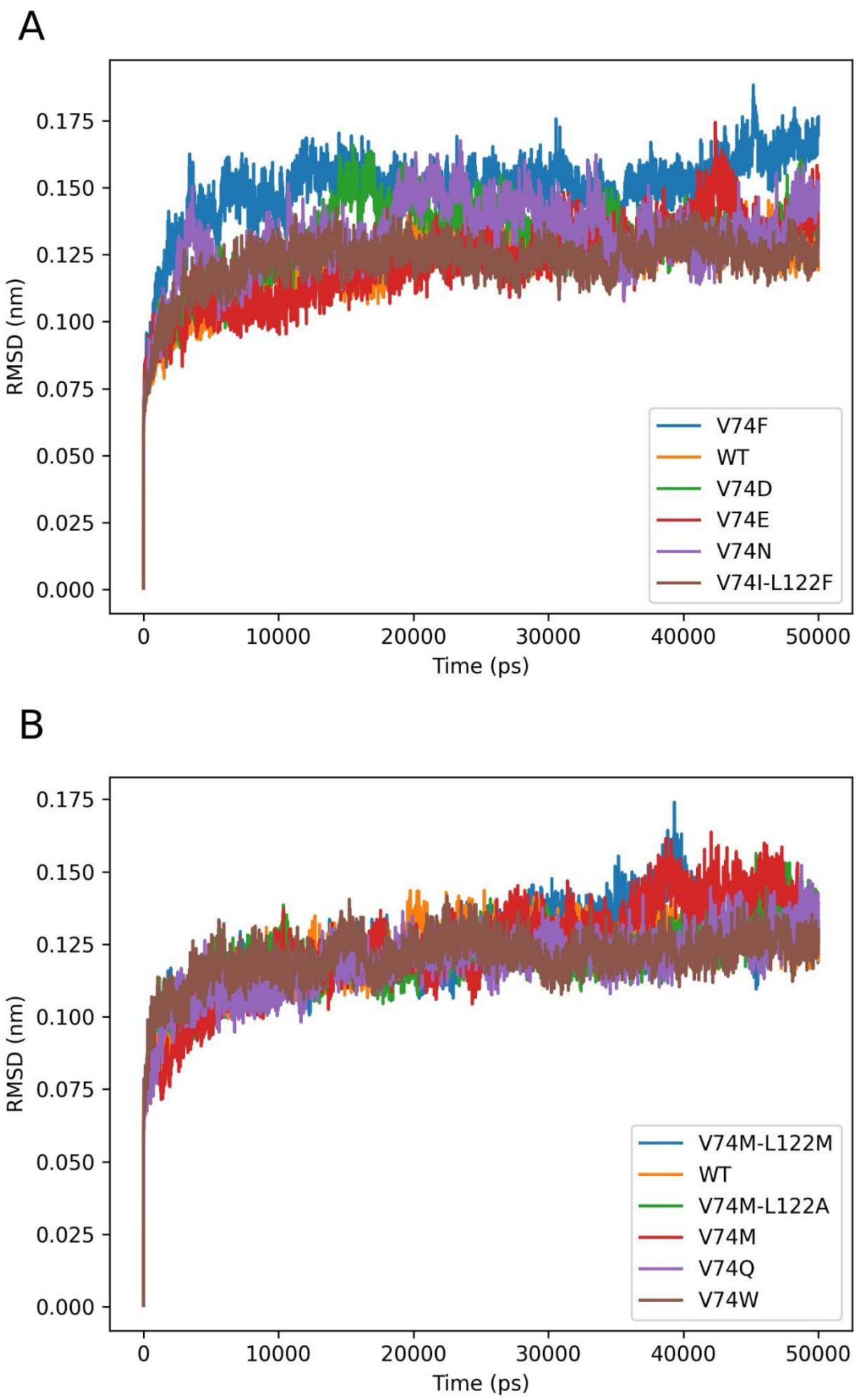

Figure S1. Backbone root mean square deviation (RMSD) values for each [NiFe] hydrogenase mutant in the 50 ns MD runs.

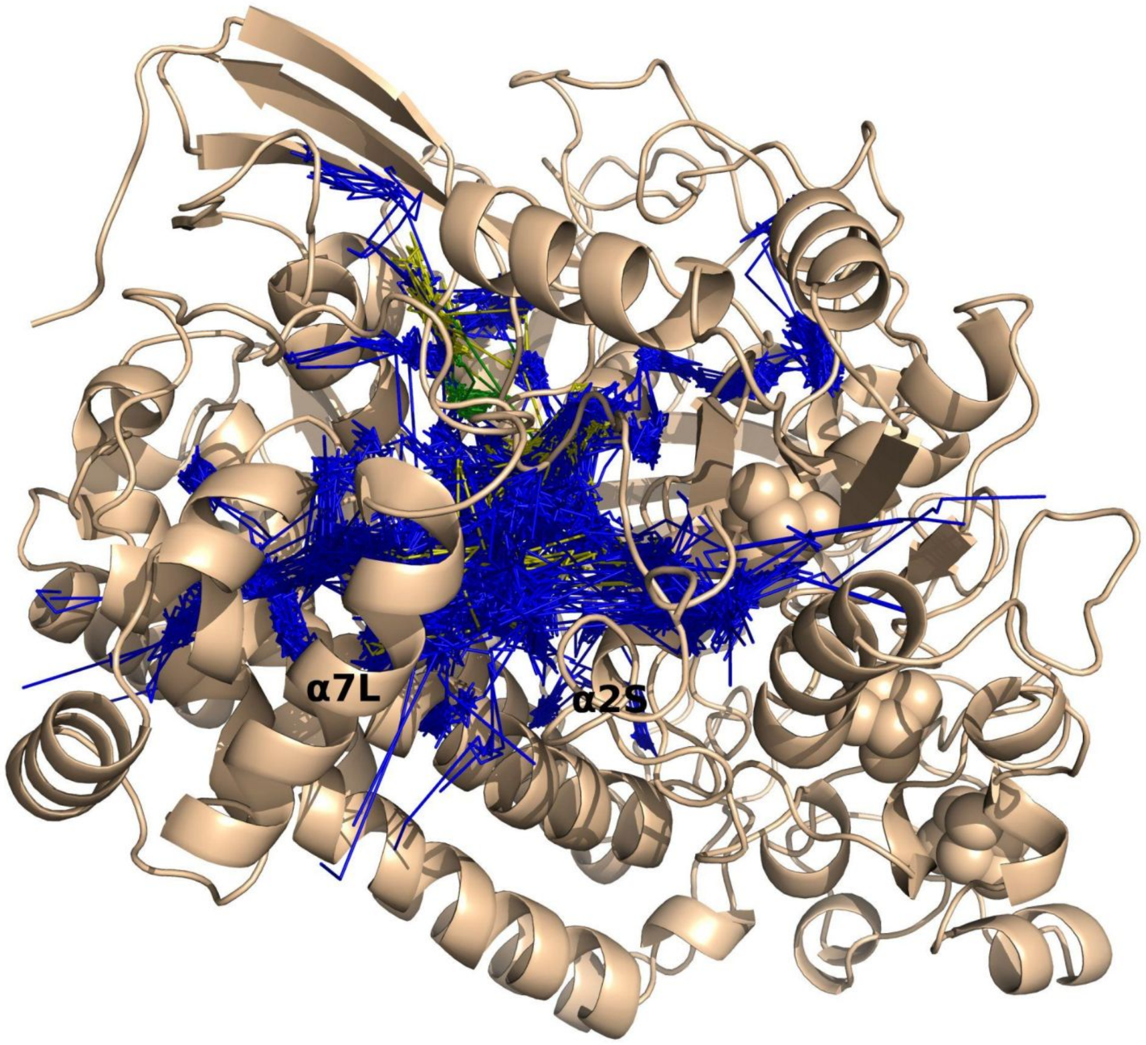

Figure S2. The unbinding pathways (blue lines) of CO from the WT [NiFe] hydrogenase. These pathways correspond to the raw pathways identified by AQUA-DUCT in the 75 unbinding trajectories.

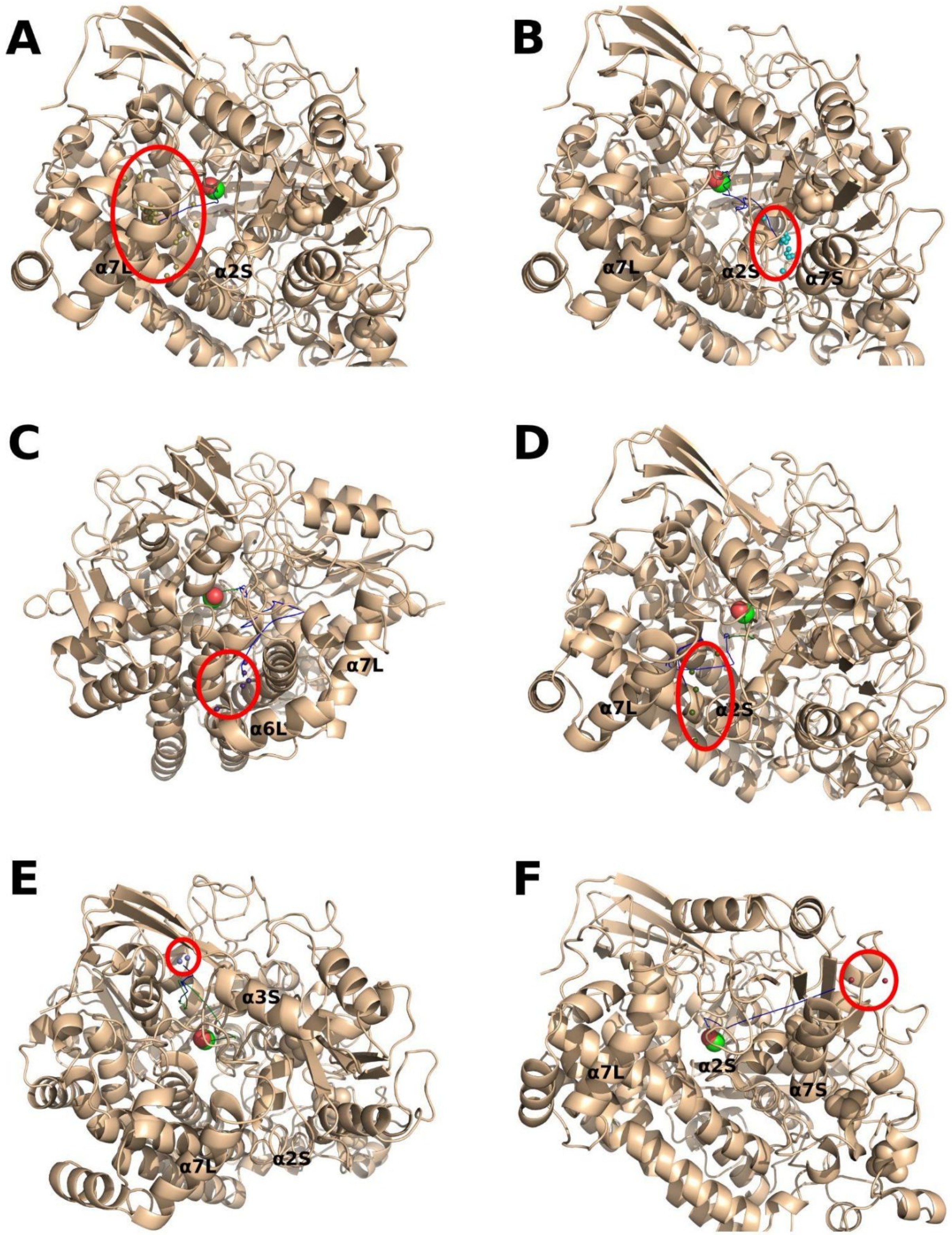

Figure S3. Description of the assignment of pathways to tunnels for the WT [NiFe] hydrogenase using AQUA-DUCT. First, AQUA-DUCT was used to identify and cluster the unbinding

pathways of 75 trajectories. For the WT hydrogenase, 12 clusters were obtained. The first 6 clusters had a population larger than 1 trajectory (displayed in the figure), and the other clusters had a population of one trajectory (not displayed in the figure). The average pathways are shown as blue lines and the exit points are shown as small spheres. The exit points are highlighted by red circles to enhance visualization. A) Cluster number 1 (N1), attributed to tunnel T1. B) Cluster number 2 (N2), attributed to tunnel T2. C) Cluster number 3 (N3), attributed to tunnel T3. D) Cluster number 4 (N4), attributed to tunnel T1. E) Cluster number 5 (N5), attributed to tunnel T4. F) Cluster number 6 (N6), attributed to tunnel T2. The Pymol session generated by AQUA-DUCT is available as supporting information in the Zenodo repository.

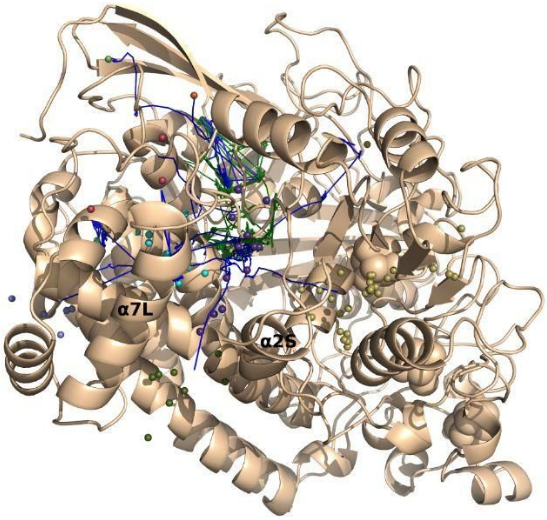

V74D

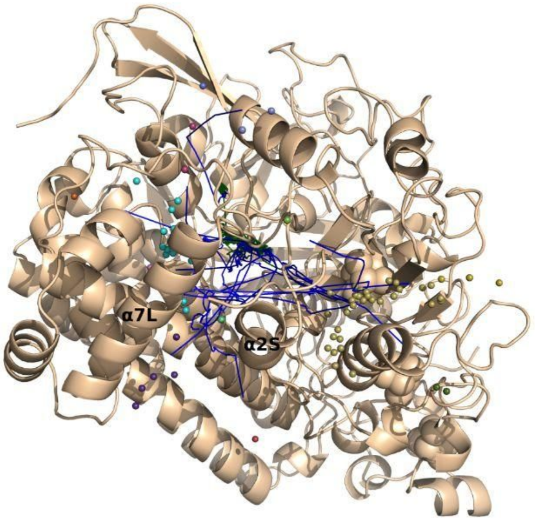

V74I L122F

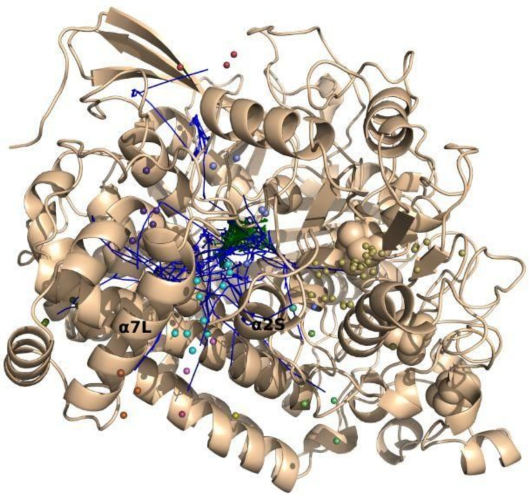

V74E

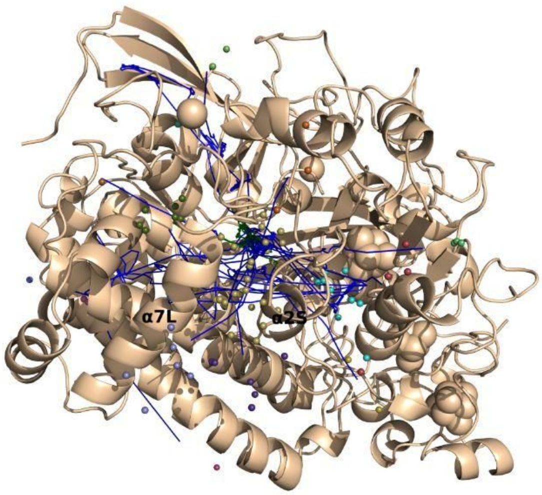

V74N

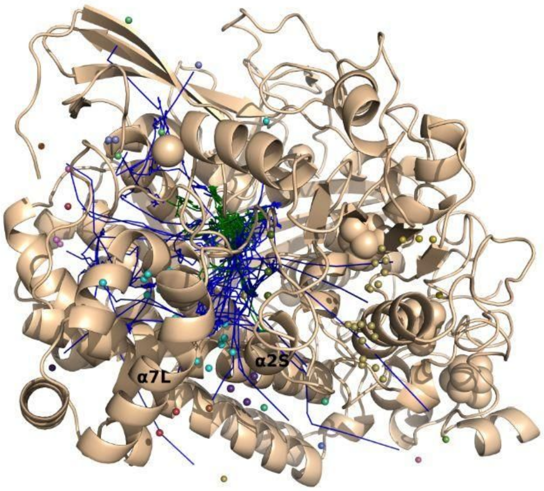

V74F

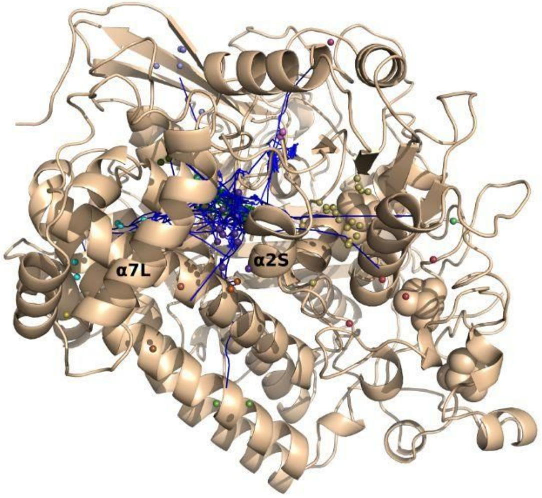

V74M L122A

Figure S4. The unbinding pathways of CO from each mutant of [NiFe] hydrogenase. The average path traces of each cluster are shown as blue lines. The small spheres represent the exit points of CO in each trajectory.

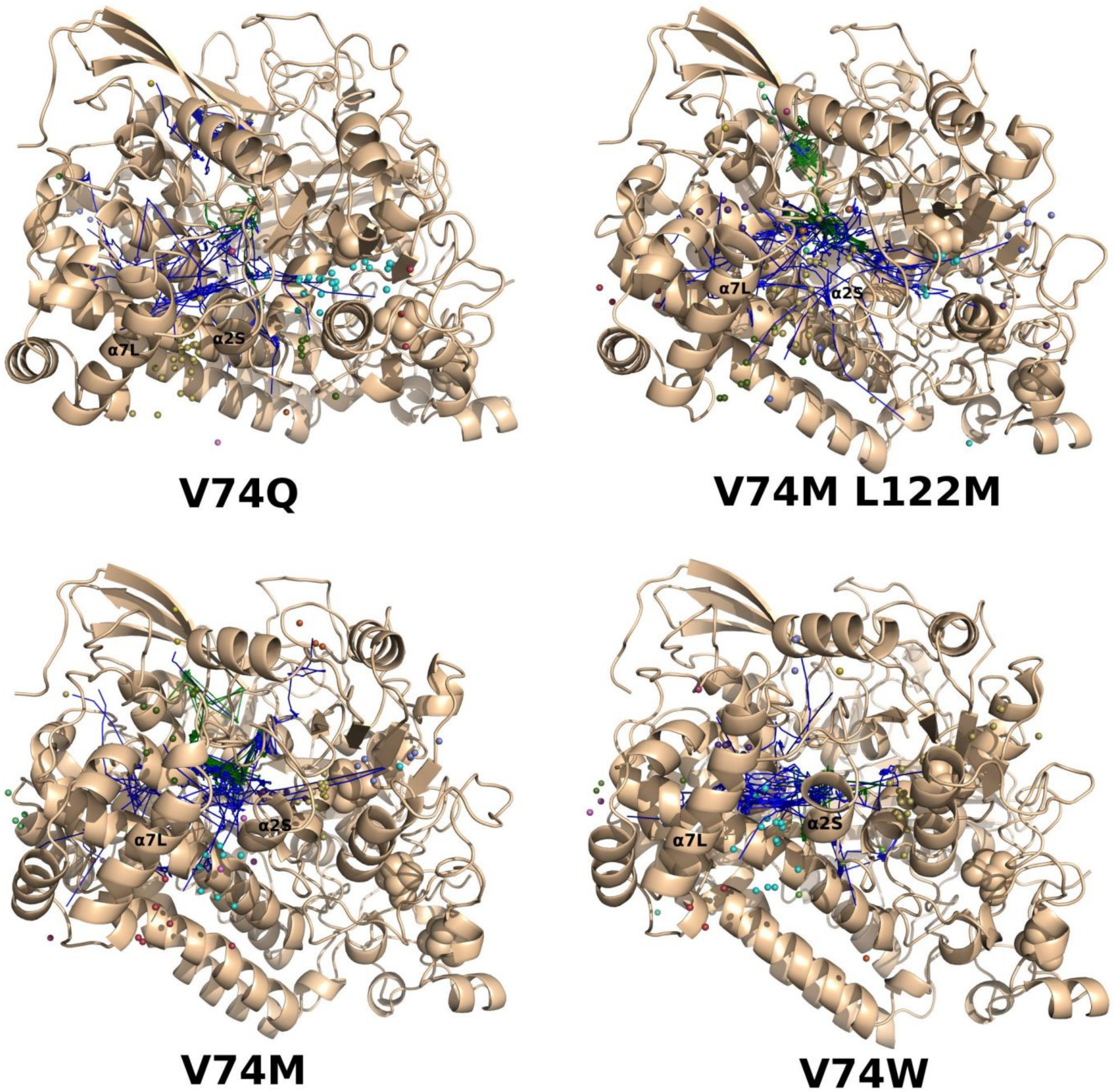

Figure S4. Continued.

τRAMD parameters optimization

There are two main parameters in τRAMD that can be optimized, (i) the magnitude of the random force and (ii) the threshold distance. The magnitude of the force has a direct effect on the residence time. As a point to start with, we checked the work of Nunes-Alves et al.[5] and found that they used a random force of 4 kcal/molÅ to obtain unbinding events for indole and benzene from

T4 lysozyme mutants. We started the tests for the magnitude of the random force from 4 kcal/molÅ and decreased it to 2, 1 and 0.5 kcal/molÅ. To find the optimum force for unbinding of CO from [NiFe] hydrogenase, we tested the [NiFe] hydrogenase-CO complex with the shortest RT, the WT [NiFe] hydrogenase-CO complex, and on the other side of the scale, the [NiFe] hydrogenase-CO complexes with the longest RTs, the complexes of CO with the V74M and V74W mutants. We obtained 75 independent dissociation events for each complex and computed the mean RT values for each mutation with different force magnitudes (Table S3 and Figure S5). Among the 4 force magnitudes we tested, we chose 1 kcal/molÅ, since it presented the best compromise between computational time required to achieve unbinding events and separation between complexes with shortest and longest RT values.

Table S3. Mean residence time (RT) values and associated standard deviations (STD) for CO dissociation from [NiFe] hydrogenase in the tests using different force magnitudes.

Force	Mutation	Mean RT (ns)[a]	STD (ns)[b]
4 kcal/molÅ	WT	0.70	0.21
	V74M	0.86	0.14
	V74W	1.28	0.25
2 kcal/molÅ	WT	2.06	0.81
	V74M	5.88	2.25
	V74W	9.06	0.73
1 kcal/molÅ	WT	5.70	3.21
	V74M	19.84	2.82
	V74W	53.62	9.62
0.5 kcal/molÅ	WT	11.86	5.49
	V74M	60.78	20.59
	V74W	93.64	16.66

a. Computed mean residence time values from τRAMD simulations.

b. Standard deviation values associated with the computed mean residence time values.

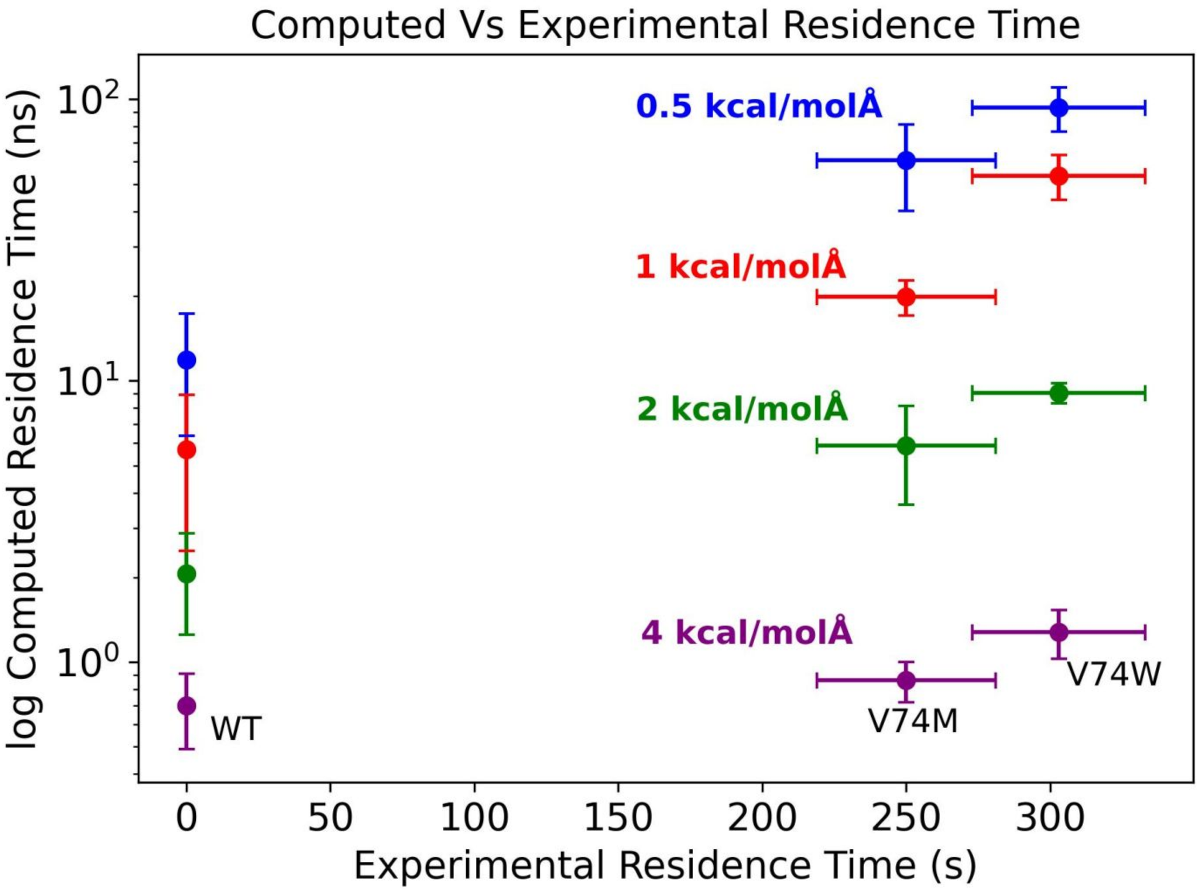

Figure S5. Comparison of experimental and computed residence times for different force magnitudes for CO dissociation from the mutants of [NiFe] hydrogenase with the shortest (WT) and the longest (V74M and V74W mutants) residence times.

The next parameter that we tested was the threshold distance. The threshold distance controls the decision of the τRAMD method to change the orientation of the force. Every 100 fs of the simulation, there is a step called the evaluation step. If the distance between the center of mass (COM) of the ligand and the COM of the protein is higher than the threshold distance, the orientation of the force will be maintained. On the other hand, if the distance is lower than the threshold distance, it is assumed that the ligand is stuck in the unbinding pathway and the orientation of the force will be changed randomly. The default value for this parameter is 0.0025 nm, and we tested double and half of this value (Table S4 and Figure S6). When we set a higher threshold distance value, the changes in the orientation of the force were more frequent, since this criteria is fulfilled less often in each evaluation step. This is the reason why with a threshold distance of 0.005 nm, the mean RT value is smaller than with 0.0025 nm, since the ligand does not stay in an intermediate state for a long time. We can also see the other face of this behavior with 0.00125 nm. With this value for the threshold distance, we observed longer RT values since the orientation of the force will not be changed as frequently as the other systems with higher values of threshold distance. We decided to use the default value for the threshold distance, 0.0025 nm, for the τRAMD simulations. Although differences in this value change the RT values, they do not change the ranking of RT values among complexes.

Table S4. Mean residence time (RT) values and associated standard deviations (STD) for CO in complex with WT [NiFe] hydrogenase or with the V74M mutant using different values of threshold distance.

threshold distance (nm)	Mutation	Mean RT (ns)[a]	STD (ns)[b]
0.005	WT	5.24	3.35
	V74M	20.26	7.48
0.0025	WT	5.70	3.21
	V74M	28.50	17.25
0.00125	WT	8.68	2.71
	V74M	37.24	6.32

a. Computed mean residence time values from τRAMD simulations.

b. Standard deviation values associated with the computed mean residence time values.

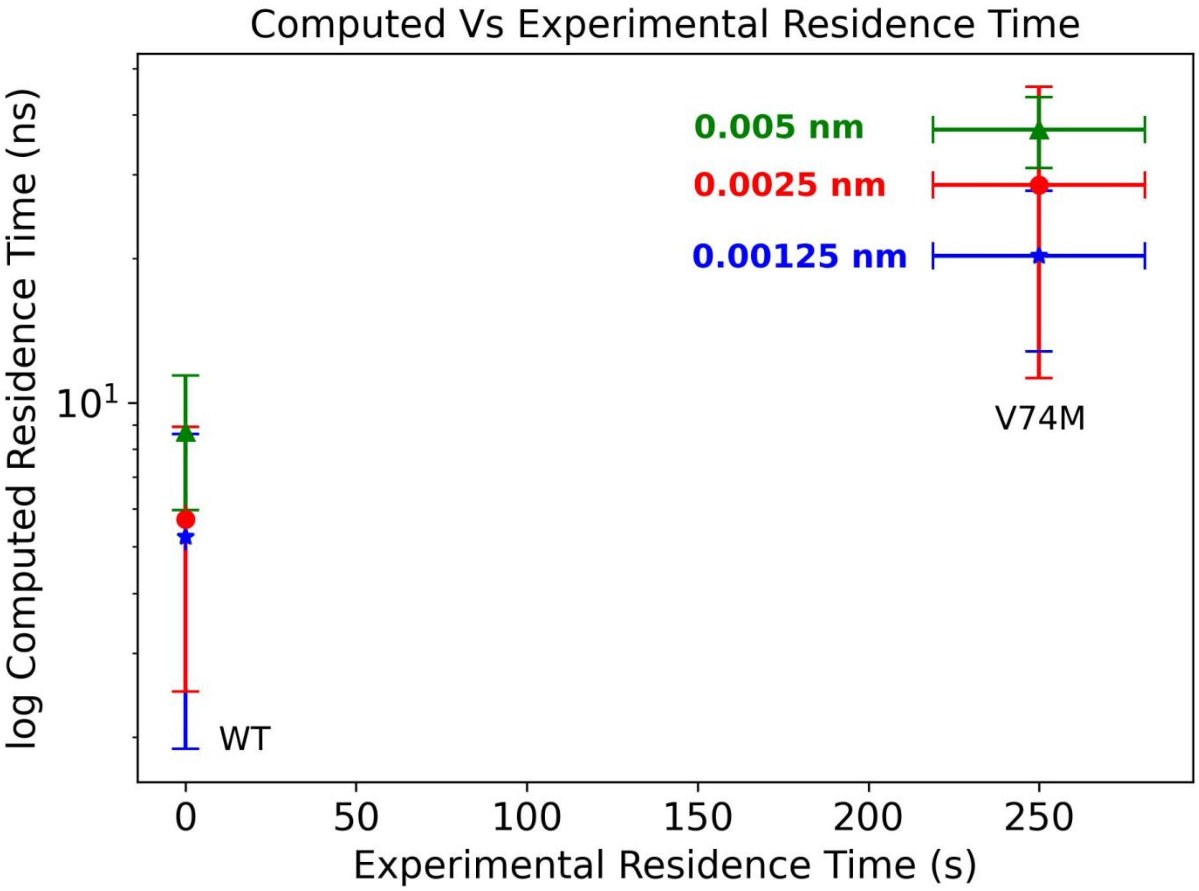

Figure S6. Comparison of experimental and computed residence times for different threshold distances for CO dissociation from the WT [NiFe] hydrogenase and from the V74M mutant.

Table S5. Pearson's correlation coefficient (R), coefficient of determination (R^2) and Spearman's rank correlation coefficient (ρ) of all data, data with outliers (V74N and V74Q) removed and data with WT removed.

	**all data**	**outliers (V74N and V74Q) removed**	**WT removed**
**R**	0.62	0.79	0.29
**R^2**	0.39	0.62	0.08
**ρ**	0.57	0.75	0.43

Distances measured between the two residues in the bottlenecks

In order to calculate the width of the bottlenecks, we calculated the distance between the side chain terminal atoms of the investigated residues and selected the lowest distance values in each frame of the simulation when there was more than one terminal atom available for the residue. The residues' atom names and numbers are presented in Table S6.

Table S6. Atom names of the residues used to calculate the distances for the 74-122 and 74-476 bottlenecks.

**Mutation**	**Residue 74**	**Residue 122**	**Residue 476**
WT	V74-CG1/CG2	L122-CD1/CD2	R476-NH1/NH2
V74D	D74-OD1/OD2	L122-CD1/CD2	R476-NH1/NH2
V74I-L122F	I74-CD	F122-CE2	R476-NH1/NH2
V74E	E74-OE1/OE2	L122-CD1/CD2	R476-NH1/NH2
V74N	N74-ND2	L122-CD1/CD2	R476-NH1/NH2
V74F	F74-CZ	L122-CD1/CD2	R476-NH1/NH2
V74M-L122A	M74-CE	A122-CB	R476-NH1/NH2
V74Q	Q74-OE1/NE2	L122-CD1/CD2	R476-NH1/NH2
V74M-L122M	M74-CE	M122-CE	R476-NH1/NH2
V74M	M74-CE	L122-CD1/CD2	R476-NH1/NH2
V74W	W74-CH2	L122-CD1/CD2	R476-NH1/NH2

Time in the tunnels

To calculate the time that CO spent in the tunnels without contact with residues 74 or 122

before it left the enzyme, we deducted the time the ligand had contact with these residues from the residence time (Table S7). On average, CO stayed about 4 ns inside the tunnels of [NiFe] hydrogenase. The trajectory frames which contained contacts with the residues 74 or 122 were identified using the "mindist" module in GROMACS and a threshold of 0.6 nm.

Table S7. Estimated time of CO's residency inside the tunnels without contacts with residues 74 or 122. Results from the dissociation simulations using 1 kcal/molÅ.

Mutation	Computed Residence Time (ns)[a]	STD (ns)[b]	Time in contact with 74 or 122 (ns)[c]	STD (ns)[d]	Time in Tunnels (ns)[e]
WT	5.7	3.21	3.06	2	2.64
V74D	12.22	4.6	8.96	3.61	3.26
L122F V74I	20.98	5.51	18.72	6.44	2.26
V74E	13.02	5.68	10.74	5.95	2.28
V74N	55.5	23.83	54.1	24	1.4
V74F	38.2	12.59	31.5	13.72	6.7
L122A V74M	21.44	11.84	16.96	11.46	4.48
V74Q	66.42	14.95	62.7	14.02	3.72
L122M V74M	23.36	8.87	18.4	7.29	4.96
V74M	19.84	2.82	10.88	3.77	8.96
V74W	53.62	9.62	50.26	8.73	3.36

a. Computed mean residence time values from τRAMD simulations using 1 kcal/molÅ.
b. Standard deviation values associated with the computed mean residence time values.
c. The mean time in which CO stayed in contact with residues 74 or 122.
d. Standard deviation values associated with the mean contact time values.
e. The values were calculated by deducting the 'time in contact with 74 or 122' from the 'computed Residence time' values.

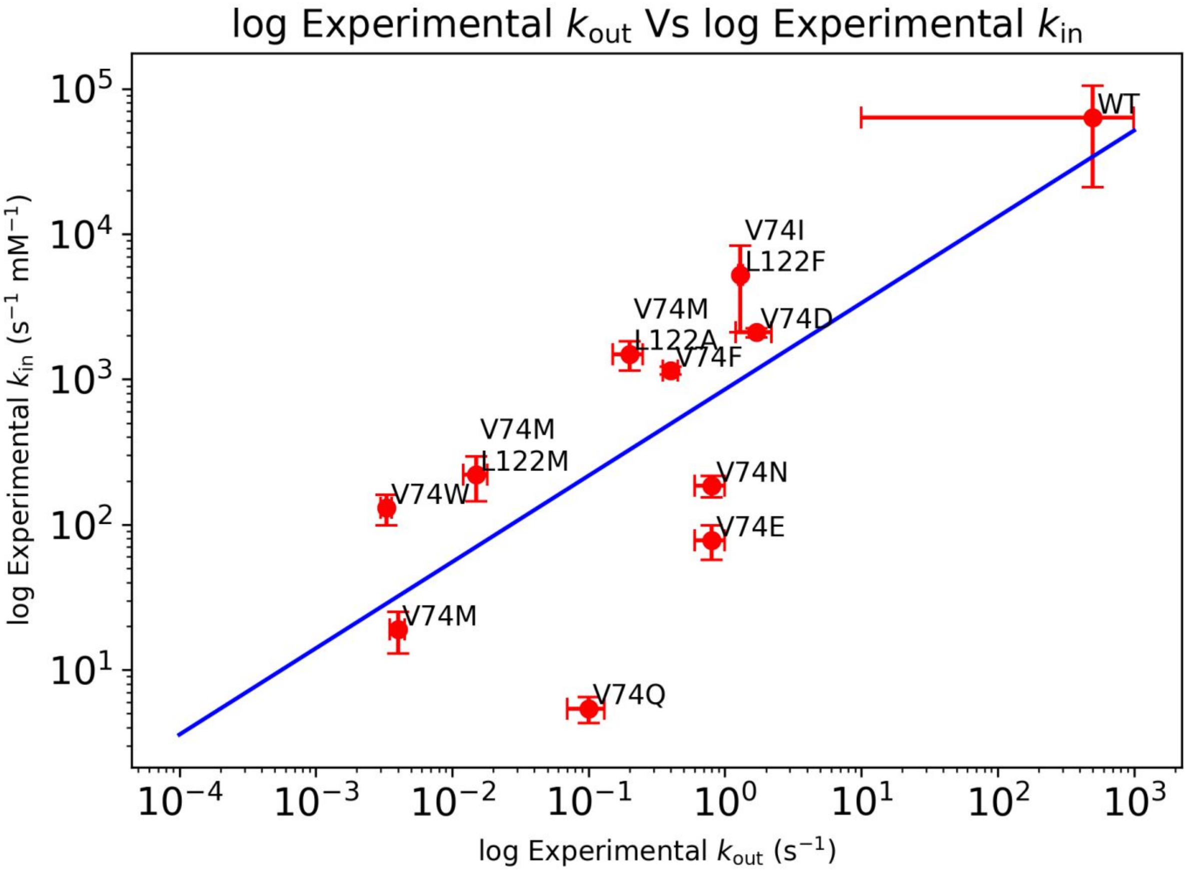

Figure S7. Comparison between experimental k_{in} and experimental k_{out} values derived from ref. 1. The high coefficient of correlation (R), 0.56, suggests that the the differences in the k_{out} values are due to differences in the transition states, rather than due to differences in the bound state.

References

(1) Liebgott, P.-P.; Leroux, F.; Burlat, B.; Dementin, S.; Baffert, C.; Lautier, T.; Fourmond, V.; Ceccaldi, P.; Cavazza, C.; Meynial-Salles, I.; Soucaille, P.; Fontecilla-Camps, J. C.; Guigliarelli, B.; Bertrand, P.; Rousset, M.; Léger, C. Relating Diffusion along the Substrate Tunnel and Oxygen Sensitivity in Hydrogenase. *Nat. Chem. Biol.* **2010**, *6* (1), 63–70. https://doi.org/10.1038/nchembio.276.

(2) Wang, S.; Hou, K.; Heinz, H. Accurate and Compatible Force Fields for Molecular Oxygen, Nitrogen, and Hydrogen to Simulate Gases, Electrolytes, and Heterogeneous Interfaces. *J. Chem. Theory Comput.* **2021**, *17* (8), 5198–5213. https://doi.org/10.1021/acs.jctc.0c01132.

(3) Straub, J. E.; Karplus, M. Molecular Dynamics Study of the Photodissociation of Carbon Monoxide from Myoglobin: Ligand Dynamics in the First 10 Ps. *Chem. Phys.* **1991**, *158* (2–3), 221–248. https://doi.org/10.1016/0301-0104(91)87068-7.

(4) Frisch, M. J.; Trucks, G. W.; Schlegel, H. B.; Scuseria, G. E.; Robb, M. A.; Cheeseman, J. R.; Scalmani, G.; Barone, V.; Petersson, G. A.; Nakatsuji, H.; Li, X.; Caricato, M.; Marenich, A. V.; Bloino, J.; Janesko, B. G.; Gomperts, R.; Mennucci, B.; Hratchian, H. P.; Ortiz, J. V.; Izmaylov, A. F.; Sonnenberg, J. L.; Williams; Ding, F.; Lipparini, F.; Egidi, F.; Goings, J.;

Peng, B.; Petrone, A.; Henderson, T.; Ranasinghe, D.; Zakrzewski, V. G.; Gao, J.; Rega, N.; Zheng, G.; Liang, W.; Hada, M.; Ehara, M.; Toyota, K.; Fukuda, R.; Hasegawa, J.; Ishida, M.; Nakajima, T.; Honda, Y.; Kitao, O.; Nakai, H.; Vreven, T.; Throssell, K.; Montgomery Jr., J. A.; Peralta, J. E.; Ogliaro, F.; Bearpark, M. J.; Heyd, J. J.; Brothers, E. N.; Kudin, K. N.; Staroverov, V. N.; Keith, T. A.; Kobayashi, R.; Normand, J.; Raghavachari, K.; Rendell, A. P.; Burant, J. C.; Iyengar, S. S.; Tomasi, J.; Cossi, M.; Millam, J. M.; Klene, M.; Adamo, C.; Cammi, R.; Ochterski, J. W.; Martin, R. L.; Morokuma, K.; Farkas, O.; Foresman, J. B.; Fox, D. J. Gaussian 16 Rev. C.01, 2016.

(5) Nunes-Alves, A.; Kokh, D. B.; Wade, R. C. Ligand Unbinding Mechanisms and Kinetics for T4 Lysozyme Mutants from τRAMD Simulations. *Curr. Res. Struct. Biol.* **2021**, *3*, 106–111. https://doi.org/10.1016/j.crstbi.2021.04.001.

(6) Chovancova, E.; Pavelka, A.; Benes, P.; Strnad, O.; Brezovsky, J.; Kozlikova, B.; Gora, A.; Sustr, V.; Klvana, M.; Medek, P.; Biedermannova, L.; Sochor, J.; Damborsky, J. CAVER 3.0: A Tool for the Analysis of Transport Pathways in Dynamic Protein Structures. *PLoS Comput. Biol.* **2012**, *8* (10), e1002708. https://doi.org/10.1371/journal.pcbi.1002708.

(7) Volbeda, A.; Martin, L.; Cavazza, C.; Matho, M.; Faber, B. W.; Roseboom, W.; Albracht, S. P. J.; Garcin, E.; Rousset, M.; Fontecilla-Camps, J. C. Structural Differences between the Ready and Unready Oxidized States of [NiFe] Hydrogenases. *JBIC J. Biol. Inorg. Chem.* **2005**, *10* (3), 239–249. https://doi.org/10.1007/s00775-005-0632-x.

(8) Magdziarz, T.; Mitusińska, K.; Bzówka, M.; Raczyńska, A.; Stańczak, A.; Banas, M.; Bagrowska, W.; Góra, A. AQUA-DUCT 1.0: Structural and Functional Analysis of Macromolecules from an Intramolecular Voids Perspective. *Bioinformatics* **2020**, *36* (8), 2599–2601. https://doi.org/10.1093/bioinformatics/btz946.
